# Characterization of Risk Profiles for Diabetic Retinopathy Progression

**DOI:** 10.3390/jpm11080826

**Published:** 2021-08-23

**Authors:** José Cunha-Vaz, Luís Mendes

**Affiliations:** 1 AIBILI—Association for Innovation and Biomedical Research on Light and Image, 3000-548 Coimbra, Portugal; lgmendes@aibili.pt; 2 Coimbra Institute for Clinical and Biomedical Research (iCBR), Faculty of Medicine, University of Coimbra, 3000-548 Coimbra, Portugal; 3 Center for Innovative Biomedicine and Biotechnology (CIBB), University of Coimbra, 3000-548 Coimbra, Portugal

**Keywords:** diabetic retinopathy, ETDRS classification, biomarkers, visual prognosis, phenotypes, personalized medicine

## Abstract

Diabetic retinopathy (DR) is a frequent complication of diabetes and, through its vision-threatening complications, i.e., macular edema and proliferative retinopathy, may lead to blindness. It is, therefore, of major relevance to identify the presence of retinopathy in diabetic patients and, when present, to identify the eyes that have the greatest risk of progression and greatest potential to benefit from treatment. In the present paper, we suggest the development of a simple to use alternative to the Early Treatment Diabetic Retinopathy Study (ETDRS) grading system, establishing disease severity as a necessary step to further evaluate and categorize the different risk factors involved in the progression of diabetic retinopathy. It needs to be validated against the ETDRS classification and, ideally, should be able to be performed automatically using data directly from the examination equipment without the influence of subjective individual interpretation. We performed the characterization of 105 eyes from 105 patients previously classified by ETDRS level by a Reading Centre using a set of rules generated by a decision tree having as possible inputs a set of metrics automatically extracted from Swept-source Optical Coherence Tomography (SS-OCTA) and Spectral Domain- OCT (SD-OCT) measured at different localizations of the retina. When the most relevant metrics were used to derive the rules to perform the organization of the full pathological dataset, taking into account the different ETDRS grades, a global accuracy equal to 0.8 was obtained. In summary, it is now possible to envision an automated classification of DR progression using noninvasive methods of examination, OCT, and SS-OCTA. Using this classification to establish the severity grade of DR, at the time of the ophthalmological examination, it is then possible to identify the risk of progression in severity and the development of vision-threatening complications based on the predominant phenotype.

## 1. Introduction

Diabetic retinopathy (DR) is a frequent complication of diabetes and, through its vision-threatening complications, i.e., macular edema and proliferative retinopathy, may lead to blindness. Diabetes is now regarded as a global epidemic. It is estimated that by 2045 there will be 629 million people worldwide affected by diabetes. Considering that a third of people with diabetes have signs of diabetic retinopathy, with 10% developing vision-threatening retinopathy, it is clearly one of the leading causes of blindness in working-age people [1].

The presence of nonproliferative retinopathy is identified by microvascular changes that are typically asymptomatic. Nonproliferative retinopathy progresses silently, without vision loss from mild to moderate to severe stages. However, the progression of nonproliferative retinopathy to vision-threatening stages, proliferative retinopathy, and macular edema with vision loss vary from individual to individual. The cumulative occurrence of rates of progression from mild nonproliferative to vision-threatening complications has been determined to be in the order of 14–16% [2,3]. However, when there is moderate to severe retinopathy then progression to complications is in the order of 58% [2]. In any case, predicting which people with nonproliferative retinopathy are at a high risk for progression to vision loss remains a challenge. 

It is, therefore, of major relevance to identify the presence of retinopathy in diabetic patients and, when present, to identify the eyes that have the greatest risk of progression and greatest potential to benefit from treatment. 

## 2. Phenotypes of Diabetic Retinopathy Progression 

When looking at the initial stages of DR, it is said that it is present when microaneurysms and small hemorrhages appear on ophthalmoscopic examination. On histopathological examination, the first vascular changes occur in the small vessels in the form of vasoregression with endothelial proliferation, pericyte damage, and the development of microaneurysms. Characteristically these initial lesions are focal and located at the posterior pole of the retina. With the progression of the disease, the capillaries of the arterial side of the retinal circulation show increased vasoregression. With cell loss and closure, the number of microaneurysms increases, and the areas of capillary closure enlarge. As the areas of capillary closure enlarge, they are seen to be crossed by the remaining enlarged capillaries, which appear to act as arteriovenous shunts, receiving the blood directly from the surrounding closed capillary net [4,5]. Recent clinical studies using optical coherence tomography angiography (OCTA) show that capillary closure occurs very early in diabetic retinal disease and is initiated in the macula [6,7]. Later on, as the disease progresses with remodeling of the retinal circulation and an altered retinal blood flow distribution, probably through preferential arteriovenous preferential channels, capillary closure develops also in more peripheral regions of the retina [8,9].

Using the examination methods now available, it may be stated that the earliest alterations that may be detected clinically in the retina in diabetes are alterations in the neurosensory retinal function, breakdown of the blood–retinal barrier, and capillary closure. These alterations can be detected before ophthalmoscopic signs of DR are visible in preclinical retinopathy [6,10].

Hyperglycemia appears to be sufficient to initiate the development of DR, as revealed by the development of retinopathy in animals experimentally made hyperglycemic. However, the observation that not all individuals with poor metabolic control develop advanced stages of DR suggests that other factors, such as environmental factors and genetic predispositions, are likely to determine individual susceptibility to the disease.

Diabetic retinopathy has been generally considered to be a microvascular complication of diabetes, limiting the diagnostic and therapeutic focus to the vascular system. However, recent evidence has been accumulating, suggesting that DR involves the neuronal as well as the vascular compartments.

The neurosensory retina has recently been shown to be altered very early in diabetes and may function as the trigger for microcirculatory changes [11]. Together with reduced corneal nerve sensation and impaired autonomic innervation of the pupil, altered function of the retinal indicates that diabetes causes denervation of multiple sensory inputs to the eye. There are, therefore, strong arguments for diabetic retina neuropathy. However, it is now clear that the micro-vascular changes occur to a different degree in different patients [6]. In fact, the direct link between neurodegeneration and microvascular disease may occur predominantly in the initial stages of the retinal disease, when different individuals respond differently to the neurodegenerative changes [12].

Regarding systemic factors, it is recognized that the duration of diabetes and the level of metabolic control determine the development of DR. However, these risk factors do not explain the great variability that characterizes the evolution and rate of progression of retinopathy in different diabetic individuals. There are many diabetic patients who, after many years with diabetes, never develop sight-threatening retinal changes, whereas other patients progress rapidly. This is a message of major relevance when dealing with type 2 diabetes.

It is now accepted that only a subset of individuals with diabetes who develop retinal changes is expected to progress to advanced retinopathy stages and is at risk of losing functional vision during their lifetime.

We have identified three major phenotypes of DR progression: One, the neurogenerative phenotype characterized by slow progression, where neurodegeneration is the only identified alteration and the retinal changes may be only a manifestation of the systemic neuropathy; a second one, the leaky phenotype characterized by the added occurrence of edema resulting from the breakdown of the blood–retinal barrier which may occur at any time in the disease progression, even in the absence of relevant microvascular pathology and, finally, a third one, the ischemic phenotype, identified by increased microaneurysm turnover and the presence of active microvascular lesions. In a series of follow-up studies of 2 and 5 years, in eyes with minimal retinopathy, the first phenotype was identified in 40% of the eyes with any evidence of retinopathy and only rarely progressed to sight-threatening complications, whereas the second phenotype representing approximately 30% of the eyes with minimal retinopathy showed a relatively high risk for development of mild and manageable macular edema, and the third representing the remaining 30% of the eyes showed the higher risk for development of both clinically significant macular edema and proliferative retinopathy [3]. 

A fundamental characteristic of DR is that its progression varies in different individuals, and the development of vision-threatening complications occurs only in a few individuals. The activity of the disease and its progression varies from patient to patient, making identification of biomarkers of progression of DR to vision-threatening complications a major need. 

Treatment options presently available are limited. In those receiving treatment, therapy is largely limited to pan-retinal photocoagulation and anti-Vascular Endothelial Growth Factor (VEGF) intravitreal injections [13]. Pan-retinal photocoagulation is, however, usually deferred until nonproliferative retinopathy becomes severe and is associated with some degree of decline in visual function. Although there is no effective treatment that prevents nonproliferative diabetic retinopathy progression, the changes occurring in the retina may regress, indicating that there is a real opportunity for drugs or other treatments to stop retinopathy progression [6]. Treatments directed at the initial stages of nonproliferative diabetic retinopathy, particularly treatments that can be administered outside of a clinical setting, such as oral or topical formulations, are particularly desirable [14].

In the present paper, we review the ETDRS current nonproliferative retinopathy classification to grade retinopathy severity of the ischemic phenotype and suggest the development of a simple to use alternative to the ETDRS grading system, which establishes disease severity as a necessary step to further evaluate and categorize the different risk factors involved in the progression of diabetic retinopathy.

The final objective is to identify in an individual patient its risk profile in order to establish a personalized program of care with timely intervention, and it is realized that an automated and simple to use a severity grading system of DR is a necessary step that will facilitate the identification of risk: markers of DR progression.

## 3. The Early Treatment Diabetic Retinopathy Study and Classification of Diabetic Retinopathy Severity

The Early Treatment for Diabetic Retinopathy Study (ETDRS) disease severity scale is based on a modified version of the Airlie House classification system. The ETDRS classification is made using stereoscopic color fundus photography obtained from seven standard fields (30 degrees) and is the reference standard for grading diabetic retinopathy severity (ETDRS Report 10). More recently, digital evaluations were demonstrated to be comparable, and these have replaced stereoscopic color photographs [15].

An alternative simplified scale, the International Clinical Diabetic Retinopathy Disease severity scale, was developed with the main objective of facilitating communication between all levels of healthcare provider, but did not replace the ETDRS classification, because the severity grades needed the classic ETDRS grading process to be substantiated and verified. It is relevant that this classification chose to separate the grading of two phenotypes, the ischemic phenotype based in the ETDRS classification and the edema of the leaky phenotype [16].

The ETDRS classification is definitely the validated gold standard for diabetic retinopathy severity classification but has major limitations; it is time-consuming and not practical for daily clinical use. There is, therefore, a clear and established need for an alternative method that can perform at least as well as the ETDRS classification and can be used in clinical practice.

An alternative ETDRS classification should be able to discriminate reliably the different severity stages of diabetic retinopathy, as well as the present ETDRS classification. It needs to be validated against the ETDRS classification and, ideally, should be able to be performed automatically using data directly from the examination equipment without the influence of subjective individual interpretation.

The final goal, again, would be to use this framework on which risk factors and specific rates of progression could be identified to be associated with increased risk of progression.

## 4. Automated Alternative ETDRS Classification

The ETDRS classification is based on the identification of a series of lesions, mostly resulting from microvascular disease, and based on their distribution in the seven fundus image fields collected (Table 1). Different grades of severity are identified by the number of quadrants of the retina showing specific lesions, such as microaneurysms and hemorrhages, intraretinal vascular abnormalities, venous loops, hard exudates, and cotton wool spots, all of them resulting from microvascular pathology (Table 2).

However, at least three main disease pathways occur in diabetic retinal disease: neurodegeneration, edema, and ischemia [6,18]. A forward-looking approach should be to develop a quantitative assessment of these three different disease pathways. Our group used a non-invasive, multimodal approach to evaluate and quantify the relative relevance of these disease pathways in eyes with nonproliferative diabetic retinopathy using OCT and OCTA. Neurodegeneration was identified by the thinning of the retinal tissue (ganglion cell layers plus inner plexiform layer—GCL + IPL), this metric comparing favorably with thinning of the retinal nerve fiber layer (RNFL) because of robustness of measurements. Retinal edema was characterized by increases in retinal thickness. Finally, ischemia was identified by microvascular metrics using OCTA.

This and a series of other studies have confirmed the relevance of vessel density metrics obtained with SD-OCTA and SS-OCTA, independently of the observer, to identify microvascular pathology occurring in the diabetic retina and their value in discriminating different ETDRS grades [6,7,10,19].

We propose that lesion distribution, which is taken into consideration in the ETDRS classification and has also been confirmed with OCTA, should be taken into account when using automated evaluation with OCTA [20,21].

Involvement of retinal mid-peripheral and peripheral regions, well demonstrated in studies with widefield fluorescein angiography, have confirmed that mid-peripheral and peripheral retinal changes need to be considered and may be essential for determining retinopathy progression, at least in the more advanced stages of DR [9,14,22]. Widefield OCTA using swept source OCTA enables the determination of the microvascular metrics over large fields of the retina [21]. Our group has been able to show that retinal capillary closure, quantified by vessel density metrics using SS-OCTA, can identify the more severe stages of nonproliferative diabetic retinopathy and discriminate them from the initial stages of NPDR. A combination of acquisition protocols, using SS-OCTA, allows discrimination between eyes with mild NPDR (ETDRS 20–35) and eyes with moderate-to-severe NPDR (ETDRS grades 43–53).

This study also showed that taking into account the lesions in different quadrants contributes to discriminating levels of retinopathy severity.

We have now tested a composite of features that can be identified and obtained directly from imaging equipment available commercially, such as OCT and OCTA, which discriminate the different ETDRS severity grades. Initial results are promising and indicate that it is possible to replace the laborious and complicated ETDRS grading based on the interpretation of fundus images by a series of metrics obtained directly from OCT and OCTA equipment (Table 3). 

**Figure 1:**
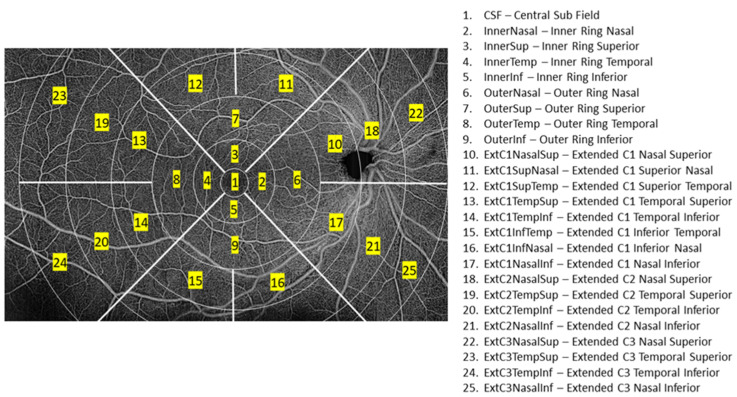
Localization of the 25 subregions that were used to decompose the 15 mm × 9 mm region acquired for each eye using the Zeiss PLEXT Elite 9000 (Carl Zeiss Meditec, Dublin, CA, USA). For each subregion, the mean value of the VD and GCL was measured. Aggregate measures were also used. The values of the inner ring (2,3,4,5), outer ring (6,7,8,9), inner circle (1,2,3,4,5), outer circle (1,2,3,4,5,6,7,8,9), and rings of the extended circles C1 (10,11,12,13,14,15,16), C2 (18,19,20,21), C3 (22, 23,24,25) were also used.

We performed the characterization of 105 eyes from 105 patients, previously classified by ETDRS level by the Coimbra Ophthalmology Reading Centre (CORC) and the object of a previous report [8] using a set of rules generated by a decision tree, having as possible inputs a set of metrics automatically extracted from SS-OCTA and SS-OCT measured at different localization of the retina (Figure 1). Data quality was checked as described in the previous work [8]. The values of the metrics were computed on the ARI network (https://arinetworkhub.com/, accessed on 1 August 2021) using the algorithms provided by the manufacture: ETDRS Retina Thickness version 0.1 (metrics related to the thickness of GCL) and the Density Quantification version 0.3.5 (metrics related with VD, PD, and FAZ).

**Figure 2:**
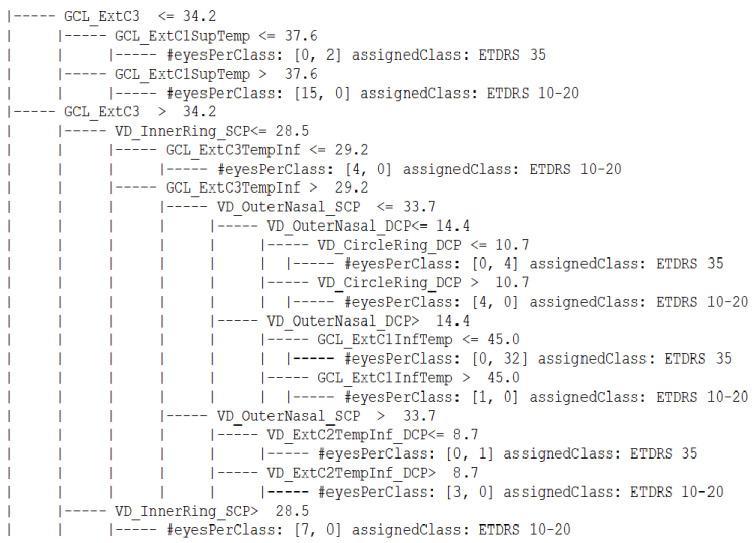
Rules derived using a CART decision tree when the task was to organize the values of the OCT and OCTA metrics extracted from the dataset comparing eyes with ETDRS 10–20 and ETDRS 35 levels. At each leaf, the number (#) of eyes selected by that rule and the most popular class (the assigned class) are presented.

**Figure 3:**
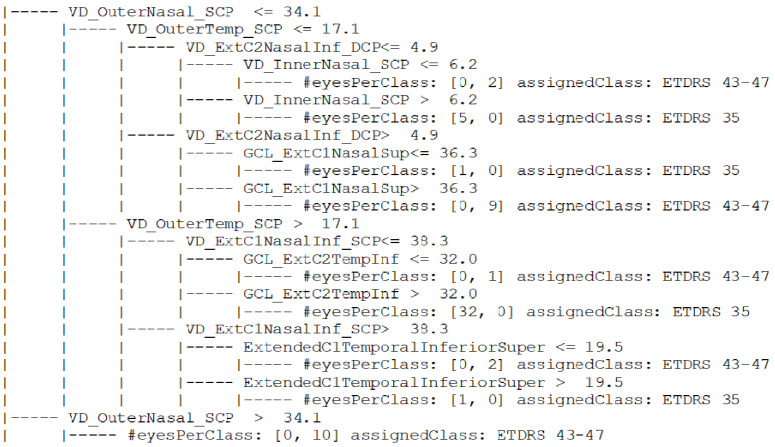
Rules derived using a CART decision tree when the task was to organize the values of the OCT and OCTA metrics extracted from the dataset when comparing with ETDRS 35 and ETDRS 43–47 level having into account the ETDRS level. At each leaf, the number (#) of eyes selected by that rule and the most popular class (the assigned class) are presented.

We started by organizing the data on rules derived from a CART (Classification and Regression Trees) decision tree between consecutive ETDRS levels. The rules were generated using the CART algorithm implemented on the python package Scikit-learn (https://scikit-learn.org/ accessed on 21 July 2021) version 0.23.2. The most important metrics included in the rules derived from the data extracted from healthy eyes (28 eyes) and eyes belonging to the ETDRS 10–20 group (34 eyes) were associated with changes in the FAZ, VD in perifovea, and thinning of the GCL + IPL localized in the mid-periphery. When the task was to organize data belonging to ETDRS 10–20 group (34 eyes) and ETDRS 35 (39 eyes), the most relevant discriminating features were associated with the VD in the central retina, extending to more quadrants and GCL thinning in the retinal mid-periphery. Between the ETDRS 35 (39 eyes) and ETDRS 43–47 (24 eyes), analysis of the most important features suggested that the VD changes were extended to the retinal mid-periphery and involved the deep capillary plexus predominately. Figure 2 and Figure 3 show the rules derived when the task was to organize the data comparing ETDRS 10–20 with ETDRS 35 and ETDRS 35 with ETDRS 43–47, respectively. When the most relevant metrics were used to derive the rules to perform the organization of the full pathological dataset, taking into account the different ETDRS grades, a global accuracy equal to 0.8 was obtained. 

An automated ETDRS classification using the data derived from OCTA and OCT equipment is, therefore, within reach. The limitations are mainly associated with the need for good quality images, which can originate from data sets with smaller sample sizes [8]. It is now necessary to collect more data using the same approach in order to apply machine learning techniques to create and validate a model to perform the ETDRS classification automatically.

The identification of IrMAs by OCTA is also considered another important step to further characterize the severity of disease before the development of PDR. It is relevant that other authors have recently attempted similar approaches [23,24].

In order to establish the progression and risk of progression, it is crucial to be able to compare examinations performed at regular intervals, and this is possible with OCT and OCTA using standardized equipment and using information given directly from the equipment. 

There is now evidence supporting the concept that DR initiates in the macular area by a deficient vascular response to abnormal neuronal toxicity caused by hyperglycemia, progressing later to the entire retina. There is initial swelling of the central retina, demanding a vascular response that is not adequate in some patients [12]. The eyes that are not able to respond adequately develop progressive capillary closure, initially around the FAZ and perifovea. This capillary closure increases as the disease progresses, with the development of collateral vascular channels and capillary closure extending to the entire retina, including mid-periphery and periphery. This capillary closure is accompanied by progressive thinning of the retina and a neovascular response that finally dominates the picture and leads to full-blown proliferative retinopathy. 

## 5. Risk Profiles of DR Progression 

In two recent follow-up studies performed by our group in different patient cohorts, one performed during a 5-year period, using OCT, and another for a period of 3-years, using both OCT and OCTA, we verified that the risk of retinopathy progression, identified by ETDRS gradings, is different between different individuals with type 2 diabetes and that ocular imaging risk markers are stronger predictors of progression to vision-threatening complications, macular edema, and proliferative retinopathy than systemic markers of metabolic control (Table 4) [23]. Furthermore, the progression of DR severity in the initial stages of DR, determined by 2-or-more-step worsening of ETDRS severity score, is associated with microvascular disease progression identified by increased microaneurysm turnover obtained from fundus photography and decreased vessel density obtained from OCTA examination [24,25].

Identifying people with the greatest risk of progression and greatest potential to benefit from treatment is clearly an important goal that appears to be now within reach using these new available methods of examination, OCT and OCTA.

Diabetic patients followed annually with OCTA and OCT show that metrics to evaluate vessel density (fovea and mid-periphery using SS-OCTA), FAZ metrics (using OCTA) to identify the ischemic phenotype, together with OCT evaluation of neurodegenerative changes (GCL + IPL thinning) can be used to identify different risk profiles of DR progression. The identification of the ischemic phenotype and leaky phenotype allows the identification of the eyes that are at risk of developing vision-threatening complications, macular edema, or proliferative retinopathy. 

The results of a three-year longitudinal study using OCT and OCTA metrics show that this goal is even more relevant when it is realized that the alterations occurring in the diabetic retina are reversible until relatively late in the disease progress and vary widely between different individuals [3]. In this three-year follow-up study, we observed that there is, indeed, marked variability in the progression of the retinal microvascular and neurogenerative changes occurring in T2 diabetes. Some patients show steady and progressive worsening, whereas others show a variable course and evidence of reversibility of their changes. These observations offer two important messages. First, the reversibility of capillary closure opens the door for early intervention with the possibility of stopping and delaying disease progression. Second, each patient should be followed closely, and risk factors should be considered to determine a specific risk profile for that patient [25].

In summary, it is now possible to envision an automated classification of DR progression using noninvasive methods of examination, OCT, and SS-OCTA. Using this classification to establish the severity grade of DR, at the time of the ophthalmological examination, it is then possible to identify the risk of progression in severity and the development of vision-threatening complications based on the predominant phenotype: ischemic, leaky, or neurodegenerative. 

## 6. Directions for Future Studies

Current nonproliferative retinopathy classifications, such as ETDRS grading, are limited by their complexity and difficulty to use in daily clinical practice. However, multimodal imaging using OCT and OCTA is offering automated alternatives based on information that can be obtained directly from the equipment and is expected to replace the laborious and complicated ETDRS classification in the near future. 

Different individuals with T2 diabetes and with the same severity degree of retinopathy show different rates of progression, both in disease severity and development of vision-threatening complications. An immediate and attainable goal will be the identification of the risk profile of each individual in order to achieve personalized monitoring of diabetic retinopathy progression and identify new tools for early intervention.

## Figures and Tables

**Table 1:** ETDRS Final Scale. Adapted from ETDRS Report No. 12 [17].

ETDRS Final Scale	
Level	Definition
10	Microaneurysms (MAs) and other characteristics absent
20	Define presence of Mas and other characteristics absent
35 A	Definite presence of venous loops in 1 field
35 B	Questionable soft exudates, Intraretinal Microvascular Abnormality (IrMA), or venous beading
35 C	Presence of Hemorrhage
35 D	Definite presence of hemorrhage in 1–5 field
35 E	Moderately severe hemorrhages in 1 field
35 F	Definite presence soft exudates in 1 field
43 A	Moderately severe hemorrhages in 4–5 fields or severe hemorrhages in 1 field
43 B	Definite presence of IrMA in 1–3 fields
47 A	Both 43 A and 43 B definitions
47 B	Definite presence of IrMA in 4–5 fields
47 C	Severe hemorrhages in 2–3 fields
47 D	Definite venous beading in 1 field
53 A	≥2 level 47 definition
53 B	Severe hemorrhages in 4–5 fields
53 C	Moderately severe presence of IrMA in 1 field
53 D	Definite presence of venous beading in 2–3 fields
53 D	Two or more level 53 definitions

**Table 2:** Basis for an alternative Diabetic Retinopathy classification.

Lesion Type (Fundus Photos)	Description	Alternative Metric	Examination
Microaneurysms and dot hemorrhages	Capillary wall outpouching (adjacent to capillary closure)	Capillary closure metrics: 1. Vessel density 2. Intercapillary spaces 3. Foveal avascular zone (FAZ) metrics	Optical Coherence Tomography (Angiography (OCT-A)
Venous beading Venous loops	Shunt vessels (?), tortuosity of venula (Adjacent to capillary closure)		
Intraretinal Microvascular Abnormalities (IrMA)	Remodeling new capillary “buds” (Adjacent to capillary closure)	Measurements performed at: 1. Perifovea 2. Mid-periphery 3. Number of quadrants involved	
Cotton wool spots	Site of ischemia, poor perfusion	Capillary closure metrics: 1. Vessel density 2. Intercapillary spaces Number of Quadrants	OCTA
Hard Exudates	Lipid and lipoproteins deposits	Central retinal thickness	Optical coherence tomography (OCT)
Neurodegenerative changes		Ganglion cell + Inner Plexiform layers thinning	

**Table 3:** Alternative Diabetic Retinopathy grading. Automated collection of parameters.

Alternative DR Grading		Perifovea	Mid-Periphery
1. Skeletonized vessel density SCP, DCP, FR	Capillary closure	✓ *	✓ *
2. FAZ area and circularity	Capillary closure	✓	
3. Thinning of GCL	Neurodegeneration	✓ *	✓ *

DCP: Deep Capillary Plexus; SCP: Superficial Capillary Plexus; FR: Full Retina; FAZ: Foveal Avascular Zone; GCL: Ganglion Cell Layer; *: number of quadrants with lesions.

**Table 4:** The five most relevant features obtained when a set of rules generated by a decision tree having as possible inputs a set of metrics automatically extracted from SS-OCTA-OCT was used to organize the data. Three different sets of rules were obtained after applying the CART method to eyes with consecutive ETDRS levels.

Control vs. ETDRS 10–20	ETDRS 10–20 vs. ETDRS 35	ETDRS 35 vs. ETDRS 43–47
Faz_Circularity	GCL_ExtC3	VD_OuterNasal_SCP
VD_CSF_Retina	VD_InnerRing_SCP	VD_OuterTemp_SCP
VD_ExtC3_DCP	GCL_ExtC3TempInf	VD_ExtC2NasInf_Deep
GCL_ExtC2NasSup	VD_OutCircle_DCP	VD_InnerNas_SCP
VD_ExtC2NasalInf_DCP	VD_EXTC1InfTemp_SCP	VD_ExtC1NasalInf_SCP

## Data Availability

Data will be available upon request to the correspondent author.
